# Early synaptic pathology is associated with small tau aggregates in Alzheimer’s disease

**DOI:** 10.1007/s00401-026-02977-9

**Published:** 2026-01-23

**Authors:** Emre Fertan, Shekhar Kedia, George Nolan, Georg Meisl, Matthew W. Cotton, Karin H. Müller, Ziwei Zhang, Leila Muresan, Annelies Quaegebeur, Maria Grazia Spillantini, David Klenerman

**Affiliations:** 1 Yusuf Hamied Department of Chemistry, University of Cambridge, Cambridge, CB2 1EW UK; 2 Dementia Research Institute at University of Cambridge, Cambridge, CB2 0AH UK; 3 Present Address: Department of Clinical Neurosciences, University of Cambridge, Cambridge, CB2 0AH UK; 4 Department of Physiology, Development and Neuroscience, Microscopy Bioscience Platform, University of Cambridge, Cambridge, CB2 3DY UK; 5 Cambridge Advanced Imaging Centre, University of Cambridge, Cambridge, CB2 3DY UK; 6 School of Computing and Information Science, Anglia Ruskin University, Cambridge, CB1 1PT UK; 7 Cambridge Brain Bank, Cambridge University Hospitals, Cambridge, CB2 0QQ UK

**Keywords:** Nanoscopic aggregates, Tau phosphorylation, Synaptic dysfunction, Super-resolution microscopy, Bayesian inference

## Abstract

Alzheimer’s disease (AD) is phenotypically characterised by progressive memory loss, which has been linked to tau aggregation and synaptic dysfunction. Here we characterised the nanoscopic tau aggregates in individual synaptosomes from AD cases and controls, measuring their number and size using SynPull with direct stochastic optical reconstruction microscopy (*d*STORM). A total of 7888 synaptosomes from pre-frontal cortex samples were studied, showing the presence of AT8-positive tau aggregates in a small fraction of synaptosomes (~ 3%) from control brains, reaching ~ 20% by Braak stage 6. These key findings of the intra-synaptic localisation of aggregates and existence of synaptic tau pathology at Braak stage 3—preceding tangle formation in this region, were confirmed using aggregate-specific single-molecule array (SIMOA) with proteinase K digestion, three-dimensional super-resolution microscopy, stimulated emission depletion microscopy (STED), and immunohistochemistry. The aggregates also grew in size with AD progression with an average length of 117 nm at stage 0, 154 nm at stage 3 and 182 nm at stage 6, however they mostly remained non-elongated (circular) with average eccentricity values remaining below 0.8. We then investigated the multi-phosphorylation of synaptic tau aggregates for AT8 and T181 and quantified their co-localisation with phosphatidylserine and CD47, synaptic “eat me” and “don’t eat me” signals respectively, along with synaptogyrin-3, which contributes to tau-mediated synaptic dysfunction. T181, phosphatidylserine, and synaptogyrin-3 co-localisation with AT8-positive tau were higher during stage 3 and CD47 was lower, indicating early synaptic pathology is associated with the formation of small tau aggregates, contributing to microglia-driven synaptic loss.

**Supplementary Information:**

The online version contains supplementary material available at 10.1007/s00401-026-02977-9.

## Introduction

Alzheimer’s disease (AD) is the globally leading cause of dementia, whose primary symptoms are cognitive impairment and memory loss [61]. While the extracellular beta-amyloid (Aβ) plaques and neurofibrillary tangles made of hyperphosphorylated tau are the neuropathological hallmarks of AD, synaptic dysfunction and loss correlate with cognitive decline more strongly than plaque/tangle formation and neuronal loss [43, 68]. Noteworthily, synaptic dysfunction has been reported to precede the Aβ plaques and neurofibrillary tangles in the AD brain and animal models [46, 74], raising the question of the temporal order of events in AD. Recently it has been proposed that the small aggregates of Aβ and tau are highly toxic. These nanoscopic aggregates are described as “diffusible” since they remain in the supernatant after a high-speed centrifugation, separating them from the fibrils making up the plaques and tangles [30, 71]. As they are capable of interacting with various receptors [28], organelles [39, 69], and nucleic acids [16, 57], they have been suggested to be the primary species promoting AD pathology.

While the presence of small-diffusible (sometimes termed “oligomeric”) tau aggregates has been documented in the synapses of the AD brain, along with fibrillar tau aggregates [65–67], the patho-physiological outcome(s) of this accumulation are not fully understood. One of the prominent theories related to the synaptic accumulation of tau is that this triggers microglial activation, which then prune the synapses and acquire a disease-associated inflammatory phenotype [55, 70]. These disease-associated microglia (DAM) then further drive AD pathology through inflammatory mechanisms [1, 40]. Another proposed disease mechanism involves the direct synaptic transmission of tau aggregates, which has been documented in mouse models [10–12] and suggested by functional-connectivity studies in humans [21, 62]. Since aggregated tau is known to have seeding activity [45], it has been proposed that the synaptic transmission of tau aggregates may cause seeding-driven aggregation in functionally connected neurons, in a “prion-like” manner [2, 24, 51]. While there is evidence for both the inflammation-driven (indirect) and seeding-driven (direct) spread of tau pathology in AD, the relative contribution of these mechanisms and their relationship with oligomeric and fibrillar tau is an active area of research. Although some studies did suggest the synaptic transmission [48] and seeding competence of oligomeric tau [47], seeding is primarily studied using fibrillar aggregates [27, 72], and it has been shown that fibrillar aggregates of tau around 170 nm in length are the most seeding-competent species [29]. Meanwhile, the smaller and rounder tau aggregates in synapses are shown to induce local inflammation through microglial activation [47], which was also shown to be induced by the presence of fibrillar tau in the neurites [8]. As such, even though neither mechanism excludes the probability of the other and ongoing studies provide evidence that both are likely to occur in the same brain, these pathological outcomes of synaptic tau aggregate accumulation could be associated with different types of tau aggregates. This link between tau aggregate morphology and pathological outcome makes it crucial to further characterise the actual tau aggregates present in the synapses at different disease stages, so appropriate therapeutics can be developed targeting the “right” aggregates. Indeed, recent studies on Aβ aggregates have shown the importance of targeting the morphologically correct species for therapeutic success [18, 36].

While the evidence for the presence of different types of tau aggregates in the synaptic compartments and their role in synaptic and cellular dysfunction is growing, the small size of these aggregates (which is mostly below the diffraction-limit of light) along with their low abundance, and high heterogeneity of their morphology and post-translational modifications makes them challenging to detect and characterise at the single-synapse level. Our group has developed advanced single-molecule detection tools using super-resolution microscopy [5, 15, 17] and digital-ELISA[6] based methods to quantify and characterise the small aggregates formed in neurodegenerative diseases and we have recently extended these techniques to synaptosomes harvested from post-mortem human brains and mouse models. This technique, called SynPull, combines single-molecule pulldown and single-molecule microscopy to quantify and characterise small aggregates inside individual synaptosomes [31]. Using SynPull, we have investigated the Aβ and AT8-positive [23] tau aggregates in late-stage AD [31] and mouse models, as well as alpha-synuclein aggregates in Parkinson’s disease brain samples and mouse models [31]. These analyses demonstrated that small tau aggregates are the dominant species in the pre-synaptic compartment in AD, indicating their role in synaptic pathology.

In the current study, we utilised SynPull to investigate synaptic tau aggregation at different stages of AD (Braak stage 0, 3, and 6) in human post-mortem pre-frontal cortex samples, characterising the synaptic and extra-synaptic AT8-positive tau aggregates throughout the disease progression and particularly at early stages. Braak stages 3 and 6 were chosen to study the synaptic alterations prior to (Stage 3) and during (Stage 6) the presence of tau tangle pathology in the pre-frontal cortex [7] to investigate the temporal order of small tau aggregate accumulation in the synapse and insoluble tangle formation. Using direct stochastic optical reconstruction microscopy (*d*STORM), we were able to quantify and characterise individual aggregates inside synaptosomes, in terms of their size and shape, providing accurate data on their morphology at single-aggregate and single-synapse level. Then we investigated multi-phosphorylation of tau at the signal synapse level by co-localising AT8 and T181 phosphorylation. Lastly, we quantified the co-localisation of (patho)physiological markers phosphatidylserine [63], CD47 [41], and synaptogyrin-3 [49] with AT8-positive tau in individual synaptosomes, determining the probability of finding these markers alongside nanoscopic tau aggregates in synaptosomes. Although our previous results did not show significant Aβ aggregate accumulation in AD synapses, diffusible Aβ aggregates can still alter synaptic physiology through proteomic changes and by inducing the translocation of tau to the synapse [20, 60]. As such, we have also quantified the diffusible Aβ levels in the same brain samples to investigate their possible association with synaptic tau pathology (Supplemental Fig. 4a&b). Collectively, our results suggest that synaptic tau aggregation starts early in AD by Braak stage 3, preceding neurofibrillary tangle formation in the pre-frontal cortex, but occurs during the presence of small-diffusible Aβ aggregates, and progresses with disease stages, with the majority of the aggregates remaining non-fibril-like. Moreover, the presence of tau aggregates in individual synapses alters the presence of markers associated with synaptic dysfunction and loss, linking small tau aggregates to microglia-driven synaptic pathology in AD.

## Methods

### Human brain samples

15 post-mortem orbito-frontal cortex (Brodmann’s area 10–11) samples acquired from the Cambridge Brain Bank were used in the experiments, with neurofibrillary tau tangle AD pathology at Braak stages 0, 3, and 6 (5 samples per stage; Table 1). Upon arrival at the Brain Bank, brains were bisected and one half was flash-frozen and stored at -80 °C while the other half was fixed in 10% neutral buffered formalin for 14 days. The formalin-fixed tissue was embedded in paraffin blocks and cut into 10 μm sections for immunohistochemical characterisation with anti-phosphotau (Ser202, Thr205) antibody AT8 [23] (Thermo Fisher Scientific, Cat. MN1020) as previously described [54]. Slide scanning at 20 × magnification was performed at the Cancer Research UK Cambridge Institute using the Aperio Scanscope AT2 (Leica Biosystems). Neuropathological examination demonstrated that Braak stage 0 cases showed an absence of AD tau pathology, stage 3 cases showed AD tau pathology not extending beyond the fusiform gyrus, and stage 6 cases showed extensive AD tau pathology extending to the primary visual cortex.

**Table 1:** Demographic data for human frontal cortex tissue samples

Case	Age	Sex	Braak stage	PMI	Cause of Death
1	69	F	Stage 0	37 h	Cardiac failure
2	65	F	Stage 0	5 h	Pulmonary fibrosis
3	73	M	Stage 0	51 h	Myocardial infarction
4	39	M	Stage 0	59 h	Cancer
5	85	F	Stage 0	36 h	Heart failure
6	86	F	Stage 3	67 h	Cancer
7	77	F	Stage 3	59 h	Lymphoma
8	85	F	Stage 3	95 h	Cancer
9	80	M	Stage 3	48 h	Cancer
10	91	M	Stage 3	23 h	Septic shock
11	91	F	Stage 6	34 h	Pneumonia
12	74	F	Stage 6	68 h	Alzheimer’s disease
13	78	M	Stage 6	44 h	Alzheimer’s disease
15	81	F	Stage 6	41 h	Alzheimer’s disease
15	71	F	Stage 6	64 h	Advanced dementia

### Synaptosome preparation

Synaptosomes were prepared as previously described [26, 31]. In brief, 300–400 mg of frozen tissue was homogenised with 1 mL of ice-cold synaptosome buffer (25 mM HEPES (pH 7.5), 120 mM NaCl, 5 mM KCl, 1 mM MgCl_2_, and 2 mM CaCl_2_, dissolved in HPLC-grade water), using a 2-mL Dounce homogeniser (Cambridge Scientific, Cat. 40,401). Then the homogenate was serially filtered through an 80 µm nylon filter (Millipore, Cat. NY8002500; 25 mm filter holder: PALL, Cat. 4320) to remove tissue debris, followed by a 5 µm filter (Millipore, Cat. SMWP04700) to remove organelles and nuclei. The product was collected in a 1.5 ml Lo-bind Eppendorf and centrifuged at 1000 g for 5 min at 4 °C. The supernatant was removed, and the pellet was reconstituted with 100 uL synaptosome buffer.

### SynPull imaging

Single-molecule pulldown (SiMPull) coverslips were prepared as previously described [15]. In brief, glass coverslips were first cleaned and passivated with polyethylene-glycol (PEG) and coated with biotinylated polyclonal anti-neurexin 1 (NRXN1) antibody (10 nm in TBS with 1 ug/mL BSA for 15 min; abcam, Cat. ab222806) using biotin-neutravidin interactions. Then the synaptosomes were incubated on the surface overnight at 4 °C. The next day, synaptosomes were fixed and permeabilised, and then synaptic AT8-positive tau aggregates were stained using Alexa Fluor™ 647 NHS ester labelled anti-AT8 antibody (2 nm in TBS with 1 ug/mL BSA for 30 min; Invitrogen, Cat. MN1020B). For the co-localisation analyses, additional antibodies against T181-positive tau (Abcam, Cat. ab236458), phosphatidylserine (Sigma-Aldrich, Cat. 16–256), CD47 (Invitrogen, Cat. PA5-80,435), and synaptogyrin-3 (Abcam, Cat. ab302614) labelled with Alexa Fluor™ 488 were added with the anti-AT8 antibody. Lastly, the synaptosome membranes were stained using CellMask™ plasma membrane stain (Thermo Fischer Scientific, Cat. C10045) for 10 min (1:6000 by volume in TBS), followed by four rounds of washes with TBS. Direct stochastic optical reconstruction microscopy (*d*STORM) imaging for super-resolution size and shape analyses [31] along with diffraction-limited imaging [5] were performed on a purpose-built total internal reflection fluorescence (TIRF) microscope [37].

### SynPull image analysis

Super-resolution images acquired using *d*STORM were analysed using the Aggregate Characterisation Tool (ACT) [73] as described previously [31]. Briefly, image preprocessing involved thresholding, dilation, and erosion to segment single molecules. The positions of segmented molecules were determined using ThunderSTORM [53], and super-resolved images were generated by superimposing Gaussian-blurred localisations with a precision of 20 nm. Synaptosomes were segmented from CellMask images using a custom pipeline, as described previously [31]. Prior to synaptosome imaging, TetraSpeck fluorescent beads (Thermo Fisher Scientific, Cat. T7279, diluted 1:1000 in PBS) were imaged in the same field of view (FoV) across both the CellMask (Thermo Fisher Scientific, Cat. C10045; 561 nm) and the antibody (640 nm) channels. These reference images were used to compute an affine matrix, which was subsequently applied to the CellMask images to correct for aberration-induced channel misalignment.

The corrected CellMask images were thresholded and regions with intensities exceeding the mean plus two standard deviations within the FoV were identified as objects of interest. Noise-induced small objects were removed by erosion and dilation operations. The remaining objects were classified as synaptosomes following size filtering as described previously [31, 33, 34]. A density-based clustering algorithm (DBSCAN) with a 75 nm epsilon distance and a minimum of two points was applied to the super-resolution localisations in the 640 nm channel to identify synaptic and extra-synaptic aggregates.

Diffraction-limited fluorescence microscopy images were acquired for multiple targets of interest, each visualised in separate channels (488 and 638 nm). Individual fluorescent puncta were segmented in each channel, and their centroid coordinates were extracted using a previously established method [31]. Using custom MATLAB scripts (R2024b, MathWorks), pairwise Euclidean distances were computed between puncta in different channels. For each punctum, the nearest neighbour distance (NND) to a punctum in another channel was calculated. A distance threshold of 50 nm was applied. A punctum was considered colocalised if its NND to a punctum in another channel was less than or equal to this threshold. For each FoV, we quantified how many puncta met this criterion, for example, the number of 561 puncta near 638, and those simultaneously near both 638 and 488.

### Extra-synaptic aggregate denaturation

To confirm that the intrasynaptic aggregates studied by SynPull are positioned inside the synaptosomes and not on the outer surface of the membrane, synaptosomes were incubated with 5% proteinase K (New England Biolabs, Cat. P8107S) at 37 °C for 30 min, followed by an incubation with 1 mM phenylmethylsulfonyl fluoride (PMSF; Cell Signalling Technology, Cat. 8553S) dissolved in isopropanol, on ice for 10 min to stop the reaction. Then the synaptosomes were lysed by adding 100 µL lysis buffer consisting of PBS (Thermo Fisher Scientific, Cat. 10,010,023) with 1% Triton X-100 (Merck, Cat. X100-100ML), protease (cOmplete™, Mini Protease Inhibitor Cocktail, Roche, Cat. 11,836,153,001) and phosphatase (PhosSTOP™, Roche, Cat. 4,906,845,001) and incubating on ice for 30 min, followed by centrifugation at 14,000 G for 10 min at 4 °C. The samples were then tested on AT8 + tau aggregate-specific single-molecule array (SIMOA) as described before [6].

### 3D Super-resolution with wide-field imaging

Three-dimensional (3D) super-resolution imaging of AT8-positive tau in the synaptosome samples was performed using the double-helix (DH) point spread function technique to obtain precise 3D localisation information. Wide-field imaging of synaptosomes was carried out in parallel to validate the spatial correspondence between the aggregates and synaptosome regions labelled with CellMask.

3D imaging was conducted on a custom-built microscope operating in highly inclined and laminated optical sheet (HILO) mode, based on an inverted microscope body (Eclipse Ti-2, Nikon). The excitation path was equipped with two laser lines, 638 nm (Cobolt, Cat. 06-MLD-180; Oxxius, Cat. LBX-638–180,) and 561 nm (Cobolt, Cat. 06-DPL-20). Both beams were expanded and collimated using Galilean beam expanders and then combined using dichroic mirrors. A 60 × water-immersion objective (NA 1.27, Nikon, Cat. MRD07650) was used for both illumination and fluorescence collection. HILO illumination was employed to minimise out-of-focus background. Fluorescence emission was separated into two detection paths using a dichroic mirror (Semrock, Cat. FF624-Di01) and recorded by two EMCCD cameras (Evolve 512 Delta, Photometrics), yielding a final pixel size of 210 nm. For 3D DH-STORM imaging, 6000 frames were acquired per condition at 20 ms exposure. For wide-field synaptosome imaging, 50 frames were acquired at 50 ms exposure.

Single-molecule localisations were first detected in the raw DH-STORM image stacks using the PeakFit function of the GDSC SMLM Fiji plug-in. The identified 2D peaks were paired and converted into 3D coordinates, and then refined and filtered using the DHPSFU plug-in (Fiji). For synaptosome analysis, the 50-frame wide-field stack was averaged to generate one image per FoV. Synaptosome regions were segmented by applying an intensity threshold of 600–65,535 and a size filter of 25–∞ pixels. A 2D binary mask was produced, and the centroid of each identified synaptosome was calculated. This binary mask was subsequently used to spatially filter the 3D *d*STORM localisations. Localisations falling within each synaptosome region were assigned accordingly, whereas those outside were excluded. The centroid of each aggregate within a synaptosome was then computed, and the 2D distances between aggregate centroids and their corresponding synaptosome centroids were measured and averaged to quantify their spatial relationship.

### Tissue sectioning, immunochemical staining, and STED imaging

Flash frozen tissue samples (same ones used in SynPull) were kept in cryovials on dry ice for cryo-sectioning. Simport Base molds were filled with optimal cutting temperature (OCT) compound (Thermo Fisher Scientific, Cat. 15,212,776) and frozen tissue pieces were placed in the molds, covered with more OCT and rapidly re-frozen in liquid nitrogen vapour. Tissue blocks were mounted on cryostat chucks using OCT and equilibrated in the cryostat for 30 min before sectioning. Ten micrometre sections were cut in a Leica CM3050 cryostat using MX35 Premier cryostat blades. Chamber temperature was set to −24 °C and specimen temperature was set to −15 °C. Sections were placed on Superfrost PLUS slides (Epredia, Cat. 4951PLUS-001) and stored in slide boxes kept on dry ice. Blocks were removed from the chucks, wrapped in tin foil and put on dry ice until storage at −80 °C.

Following cryo-sectioning, a dual immunofluorescence assay was performed to visualise AT8 + tau and synapsin (Synaptic Systems, Cat. 106–004) as the pre-synaptic marker. Slide-mounted tissue sections were post-fixed with 4% formaldehyde and subsequently permeabilised and blocked with PBS-Saponin (0.1%) and BSA (5%; Sigma Aldrich, Cat. A7906) at room temperature in a dark humidified chamber. Sections were then processed beginning with overnight incubation at 4 °C with primary antibodies against Synapsin1/2 (1:125) and AT8 + tau (1:125) in PBS-Saponin (0.1%) and BSA (5%). The following day, endogenous lipofuscin autofluorescence was quenched using TrueBlack® (Biotium, Cat. 2700-BT), after which sections were incubated for 3 h at room temperature with secondary antibodies Abberior STAR RED (1:250; AbberiorGmbH, Cat. STRED-1001-500UG) and Alexa Fluor 594 (1:250; Life Technologies Limited, Cat: A11076) in PBS-Saponin. Finally, slides were sealed with coverslips using ProLong Diamond Antifade Mountant (Invitrogen, Cat. P36961).

Stained sections were imaged using a commercial stimulated emission depletion (STED) inverted microscope (Abberior Instruments GmbH, Göttingen, Germany). This laser scanning microscope provides multiple super-resolution channels. It is based on a fully automated Olympus IX83 microscope platform with a 100x (1.4 NA) oil immersion objective (UPLSAPO 100XO) and a high-precision Ultrasonic Stage (IX3-SSU). STED image acquisition was performed with pulsed 561 nm and 640 nm laser excitation (250 µW at 40 MHz and 1mW at 40 MHz maximum output power, respectively) and depletion at 775 nm (3W at 40 MHz maximum output power). The laser powers were adjusted to 20% and 10% of their respective total power for 561 nm and 640 nm, respectively. A depletion laser power of 8% and 7% of the total power of the 775 nm laser was used to deplete the 561 nm and 640 nm, respectively. The fluorescence signal was detected with Avalanche Photodiode Detectors (APDs) operating in single photon counting mode. Images were taken for a region of interest (750 pixels × 750 pixels) with a pixel size of 20 nm. The pinhole was set to 1.0 AU.

For dual-colour STED imaging, the same depletion laser was used in lateral depletion mode while the excitation wavelength was adapted to the fluorophore. STED was performed using a pulsed depletion laser with a 775 nm wavelength. The APD1 detector (detection 650–763 nm, excitation 640 nm) was used for Abberior Star Red, and the APD2 detector (detection 584–630 nm, excitation 561 nm) was used for Alexa Fluor 594, as previously established [32, 34].

Deconvolution was performed using Huygens Professional software (Scientific Volume Imaging, Hilversum, Netherlands) in STED mode, employing the Classic Maximum Likelihood Estimation (CMLE) algorithm. Default settings were applied, with a maximum of 40 iterations. STED images of the presynaptic marker Synapsin1/2 were utilised to delineate synapses from adjacent regions. Synapse and p-tau aggregate detections were performed using the à trous wavelet pipeline implemented in the Fiji ThunderSTORM plugin [53]. Synapse candidates were filtered by applying an intensity threshold of > 200 ADU and a Gaussian standard deviation (σ) in the range between 80 and 800 nm. This filtering criterion was chosen based on the fluorescent puncta characteristics appropriate for detecting synaptic structures, as estimated from synaptic dimensions previously reported [25, 35].

The identified pre-synapses were subsequently used for analysing AT8 + tau clusters. Aggregates were modelled as anisotropic 2D Gaussian shapes using custom MATLAB scripts (R2024b, MathWorks). To identify tau aggregates in proximity to pre-synapses, a distance threshold of 90 nm was applied. An aggregate was considered colocalised if its distance to a pre-synapse in the corresponding channel was less than or equal to this threshold. These were classified as presynaptic aggregates. For each tau aggregate, the length (2.3σ long axis) was quantified.

### Tissue homogenisation

For the Aβ ELISA and SIMOA analyses, human brain samples were homogenised as previously described [31]. In brief, ~ 400 mg of tissue samples were placed into 2 mL Eppendorf protein Lo-bind tubes prefilled with 1 mm zirconium beads (Scientific Labs, Cat. SLS1414) and mixed with 700 uL of homogenising buffer (10 mM Tris–HCl, 0.8 M NaCl, 1 mM EGTA, 10% sucrose, 0.1% sarkosyl, Pefabloc SC protease inhibitor, and PhosSTOP phosphatase inhibitor tablet; pH ∼ 7.4) and the samples were mechanically homogenised on an electronic tissue homogeniser (VelociRuptor V2 Microtube Homogeniser, Scientific Labs, Cat. SLS1401) at 5 m/s for two cycles of 15 s, with a 10 s gap in between, followed by centrifugation at 21,000 g for 20 min at 4 °C. Then the supernatant was collected and stored in a protein LoBind Eppendorf at 4 °C, while the pellet was homogenised again by adding an additional 700 uL of buffer and using the same parameters. The supernatant from this step was mixed with the one from the previous step, aliquoted, and stored in a −80 °C freezer. Aβ42 ELISA (Thermo Fisher Scientific, Cat. KHB3441) was run following the manufacturer’s instructions, simultaneously with the Aβ aggregate-specific SIMOA as described previously [6, 18].

### Statistical analyses

The R Project Statistical Computing version 4.4.2 (2024–10-31)—"Pile of Leaves" was used for statistical analyses, and the graphs were generated in GraphPad Prism 7.0a for Mac OS X. *d*STORM images were acquired from 4 FoV’s (technical replicates) at 50 ms exposure for 4000 frames and diffraction limited images were acquired from 16 FoV’s at 50 ms exposure for 50 frames. Differences between groups were determined using 95% confidence intervals (CI) [52], presented in Table 2. The novel code generated for the analysis of STED data can be accessed at https://github.com/lemur01/AggregateStats and the datasets generated and analysed during the current study are available from the corresponding author on reasonable request.

**Table 2:** Statistical analysis for the comparison of the samples from different Braak stages. 95% confidence intervals of group differences are calculated and a difference not containing 0 is considered a statistically meaningful (significant) difference

95% confidence intervals	Stage 0 vs 3	Stage 3 vs 6	Stage 0 vs 6
Tau synaptic fraction	2.85, 17.05	0.87, 15.07	10.82, 25.02
Synaptic tau count	−2.80, 19.20	−6.30, 15.70	1.90, 20.90
Synaptic tau length	−18.30, 90.84	4.64, 51.68	11.00, 117.88
Extra-synaptic tau count	18.80, 144.40	157.70, 283.30	239.30, 364.90
Extra-synaptic tau length	−8.70, 2.77	−2.90, 4.40	−7.36, 2.94
Total T181	3.33, 11.39	−2.34, 5.72	5.02, 13.08
T181 synaptosome fraction	6.31, 18.72	−3.88. 8.54	8.64, 21.06
T181 + AT8 / AT8 + synaptosomes	2.41, 20.13	−3.11, 14.61	8.21, 25.82
T181 + AT8 / All synaptosomes	0.83, 9.80	1.51, 10.47	6.83, 15.79
Total PS	32.52, 47.23	−23.79, −9.09	16.09, 30.79
PS synaptosome fraction	31.53, 48.28	−18.58, −1.82	21.33, 38.08
PS + AT8 / AT8 + synaptosomes	21.52, 45.69	−17.27, 5.55	15.84, 39.64
PS + AT8 / All synaptosomes	8.47, 23.15	−11.76, 2.92	4.05, 18.73
Total CD47	−16.98, −10.70	−5.35, 0.93	−19.19, −12.91
CD47 synaptosome fraction	−27.10, −8.65	−12.06, 6.38	−29.94, −11.49
CD47 + AT8 / AT8 + synaptosomes	−27.72, 3.95	−21.90, 8.91	−34.78, −1.98
CD47 + AT8 / All synaptosomes	−0.06, 8.04	−6.76, 1.33	−2.77, 5.32
Total SYNGR3	19.43, 31.23	2.31, 14.11	27.64, 39.44
SYNGR3 synaptosome fraction	19.06, 38.51	−1.95, 17.50	26.83, 46.28
SYNGR3 + AT8 / AT8 + synaptosomes	15.93, 50.92	−17.94, 13.91	16.52, 46.29
SYNGR3 + AT8 / All synaptosomes	1.91, 15.24	−3.24, 10.09	5.33, 18.66

### Length distribution analysis

We have developed a mechanistic model for the aggregate size distribution, based on the balance of elongation and aggregate removal [13]. As our model describes the steady state length distribution of aggregates well above the nucleation size, we focus on aggregates with more than ${n}_{min}$ = 500 monomers in our analysis. To avoid biases due to individual outlier fibrils, we also set an upper limit of ${n}_{max}=1200$ monomers, as above those lengths the distribution becomes severely under sampled. We convert the aggregate lengths to monomers by multiplying the length of aggregate in nanometres by 4, from the assumption of a double stranded aggregate with a beta-sheet separation of 0.5 nm [19]. However, as shown by Cotton et al. [13], the results for the difference in relative removal rate between synaptic and extra-synaptic aggregates is insensitive to this conversion factor. Our results are thus robust to the exact conversion and similarly apply if the aggregates differ in structure from those seen in cryo-EM have a different number of monomers per nm. We use the following normalised distribution for the probability of an aggregate of a length ${\mathrm{n}}$ as

1 $$\begin{array}{*{20}c} {P\left( {aggregate \;of \;length \;n} \right) = \frac{{1 - {\upalpha }}}{{{\upalpha }^{{n_{min} }} - {\upalpha }^{{n_{max} + 1}} }}{\upalpha }^{n} , } \\ \end{array}$$

where ${\upalpha }$ is the decay length to be determined from the data and is related to the relative rate of removal.

To extract the value of α most consistent with the data, we perform Bayesian inference, which allows us to directly determine the probability of the observed dataset from the aggregate lengths, rather than requiring binning and the calculation of a histogram. We use a uniform prior for α between 0 and 1 and use the maximum likelihood value of α to plot the “best fit” line long with a data histogram and to calculate additional parameters such as the difference in relative removal rate between intra and extra synaptic aggregates below. We perform this analysis for the intra- and extra-synaptic aggregate length distributions separately, across all Braak stages, to determine ${\upalpha }_{intra}$ and ${\upalpha }_{extra}$. We define the relative removal, $~\tilde{r}$as the removal rate/elongation rate. We determine the ratio of the synaptic and extra-synaptic relative removal using

$$\frac{{r^{ \sim } _{{\mathrm{int} ra}} }}{{r^{ \sim } _{{extra}} }}~ = \frac{{\alpha _{{\mathrm{int} ra}} ^{{ - 1}} - 1}}{{\alpha _{{extra}} ^{{ - 1}} - 1}}$$

The quoted errors are for the 68th percentile from fitting the decay lengths.

### Analysis of aggregate numbers and correlation with other markers

In order to quantify the effect of AT8-positive tau presence on the probability of encountering the other protein signal (phosphatidylserine, CD47, and synaptogyrin-3), we employed Bayesian inference. Since the data contain information on the presence and absence of a certain signal, the likelihood function is a Bernoulli distribution with unknown parameter *f*, which denotes the fraction of positive synaptosomes that one would expect to observe if infinite measurements are performed. Using a beta distribution as a prior, the resulting posterior probability of f is also beta distributed and simply given by the analytical expression:

$$P(f|data) = Beta\left( {a_{prior} + n, b_{prior} + N - n} \right)$$

where *N* is the total number of synaptosomes, and *n* is the number of synaptosomes that contain the signal.

Furthermore, when comparing two distributions, such that of synaptosomes that contain the signal, and that of synaptosomes that contain the signal given they are also containing AT8-positive tau, we can evaluate the performance of the hypothesis that they were drawn from the same distribution, i.e. that $f_{P(signal + |AT8 + )} = f_{{P\left( {signal + } \right)}}$, with the hypothesis that they were not. In other words, we can work how likely it is that the presence of AT8-positive tau has an effect on the appearance of the signal. To do so, we compute the Bayes factor *B*, which is the probability of observing the data given that the two *f* parameters are the same, over the probability of observing the data given that the two *f* parameters are independent:

$$B = \frac{{P\left( {data | M_{1} } \right)}}{{P\left( {data | M_{2} } \right)}}$$

where *M*_*1*_ and *M*_*2*_ denote the different models, i.e. *M*_*1*_ means $f_{P(signal + |AT8 + )} = f_{{P\left( {signal + } \right)}}$ and *M*_*1*_ means $f_{P(signal + |AT8 + )}$ and $f_{{P\left( {signal + } \right)}}$ are independent. For easier interpretability we convert the Bayes factor to a probability that *M*_*1*_ is correct, given by $\frac{B}{1 + B}$. Thus a 50% probability corresponds to equal probabilities for *M*_*1*_ being correct and *M*_*2*_ being correct. Note that we analyse all biological repeats together, thus implicitly make the assumptions that *f* is constant across samples of the same disease stage. If in reality *f* differs between samples, the resulting posterior would be more spread out.

## Results

**Fig. 1:**
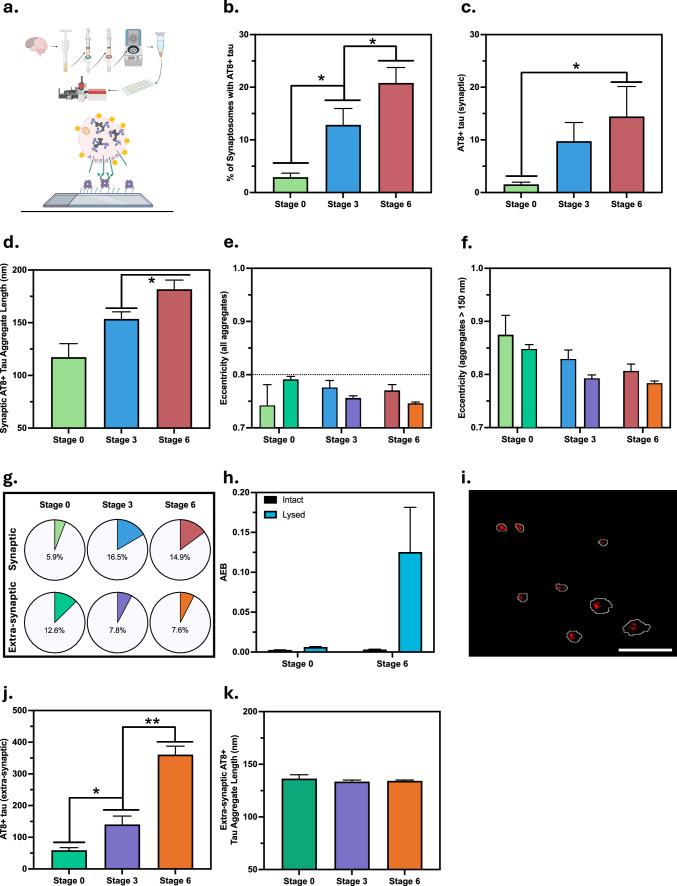
Synaptic AT8-positive tau aggregate quantity and morphology determined using SynPull. **a** Cartoon representation of SynPull methodology, showing the homogenisation, filtration, centrifugation, and imaging steps. **b** Percentage of synaptosomes containing one or more AT8 + tau aggregates, **c** number and (**d**) length of the aggregates within the synaptosomes, (**e**) eccentricity of the synaptic aggregates, indicating their shape with 0 being a perfect circle and 1 being a flat line, (**f**) eccentricity of the synaptic aggregates above a length of 150 nm. (**g**) Pie-charts showing the fraction of synaptic (top) and extra-synaptic (bottom) aggregates at each Braak stage with a length over 150 nm and eccentricity above 0.9. These aggregates are characterised as “fibril-like”. **h** Enzymes per bead (AEB) values are the SIMOA read-out for aggregate concentration in intact and lysed samples. (**i**) Three-dimensional STORM image with synaptosomes (shown in different colours) and AT8 + tau aggregates (shown in white). Error bar is 10 µm. **(j)** Number and (**k**) length of extra-synaptic aggregates at each Braak stage. Data in **b–f** and **j–k** are represented as Mean ± SEM, and differences are calculated using 95% confidence intervals (CI); a CI not including 0 is considered significant, indicated with an asterisk

To characterise the small tau aggregates positive for AT8 phosphorylation (referred to as AT8 + aggregates), we used SynPull (Fig. 1a) on the AD prefrontal cortex samples. Developed by our group [31], SynPull combines single-molecule pulldown, single-molecule chemistry, and computational analysis methods. Individual synaptosomes are harvested from frozen brain samples and anchored to a PEG-coated surface using anti-neurexin antibodies. Then they are permeabilised, and the aggregates inside, along with membrane-bound targets, are imaged using single-molecule microscopy. While SynPull can provide morphological information on aggregates below the diffraction-limit of light, it cannot determine the intrasynaptic localisation of the aggregates (versus being stuck on the outer side of the synaptosome membrane). To address this, further analyses using aggregate-specific single-molecule array (SIMOA) and three-dimensional super-resolution imaging were also performed to confirm the location of the aggregates.

### Neuropathological characterisation of the samples

We aimed to compare synaptic tau aggregation across AD disease progression using post-mortem pre-frontal cortex tissue with and without neurofibrillary tangle pathology. To achieve this, five independent cases from Braak stage 0 (control), stage 3 (early), and stage 6 (advanced) were studied (15 samples in total). While Braak stage 6 cases show advanced neurofibrillary tau tangle pathology in the pre-frontal cortex, the tau pathology in the Braak stage 3 cases is limited to the medial temporal lobe [7], which was validated by immunohistochemical analysis (Supplementary Figs. 1–3). The presence of total and aggregated diffusible Aβ in the samples was demonstrated with ELISA and aggregate-specific SIMOA [6] assays, respectively (Supplementary Figs. 4a&b).

### Synaptic tau aggregation in AD

While the presence of AT8 + tau in the synapse was not AD-specific and was also observed in control (Braak stage 0) cases, both the fraction of synaptosomes with aggregates (Fig. 1b) and the number of aggregates inside these synaptosomes (Fig. 1c) were significantly lower than AD samples. The fraction of synaptosomes containing one or more AT8 + tau aggregates showed differences between the Braak stages, with higher levels (2% versus 13%) at stage 3 than at stage 0, and even higher levels at stage 6, reaching 20% (Fig. 1b**)**. The number of aggregates per synapse showed a similar trend with an early increase at stage 3 and differed significantly between stages 0 and 6 (Fig. 1C). Along with quantifying them, we also characterised the small tau aggregates in terms of their size and shape using *d*STORM imaging (see Supplemental Fig. 5 for representative images). While the length of the synaptic AT8 + tau aggregates was also higher at later disease stages, with an average of 117 nm at stage 0, 154 nm at stage 3, and 182 nm at stage 6 (Fig. 1d), they mostly remained non-elongated (circular), with average eccentricity values remaining below 0.8 (Fig. 1e). When we focussed on the longer aggregates above 150 nm in length, the average aggregate eccentricity decreased with Braak stage (Fig. 1f). Large and elongated aggregates with a length over 150 nm and eccentricity over 0.9 remained rare, yet were significantly more common in AD cases, with a 6% occurrence rate in the control cases, 16.5% at stage 3, and 15% at stage 6 (Fig. 1g). Collectively, these results show that synaptic aggregation of AT8 + tau begins early in AD and affects more synapses as the disease progresses, accompanied by morphological changes in the aggregates as they grow in size but mostly remain non-elongated.

While SynPull enables us to quantify and characterise the aggregates associated with synapses, on its own, it cannot conclude that these aggregates are located inside the synaptosomes, as there is a chance that they may be stuck to the outer surface of the membrane. To address this, we performed two additional experiments. First, we treated the synaptosomes with Proteinase K to digest the proteins located outside. Then, the synaptosomes were either lysed or kept intact and analysed using AT8 + tau aggregate-specific SIMOA [6]. While minimal signal was observed in the stage 0 samples in both conditions, along with the intact stage 6 samples, aggregates were detected in the lysed samples from Braak stage 6, confirming the presence of AT8 + tau aggregates inside the synaptosomes (Fig. 1h). Nevertheless, this does not exclude the possibility of the existence of aggregates stuck to the outer surface of the membrane. Thus, we also performed three-dimensional super-resolution microscopy with a double-helix illumination [9]. If the aggregates were outside, we would expect to see localisations distributed around the periphery of the synaptosome, but this was not observed (Fig. 1i), providing further evidence for the intra-synaptic localisation of the aggregates. Another important control is the possibility of changes in the aggregate quantity or morphology, along with synaptic contamination during synaptosome preparation. To address these, we performed stimulated emission depletion (STED) microscopy on intact tissue slices from the Braak stage 0 and 3 samples (Supplemental Fig. 6a). While the percentage of synapses containing AT8 + tau aggregates increased from 3.1% to 17.1% at stage 3 (Supplemental Fig. 6b), the average aggregate length also grew (Supplemental Fig. 6c). While these results agree with SynPull, aggregate sizes provided by STED were overall larger, which may be due to differences between the sample preparation or imaging method for the two techniques.

**Fig. 2:**
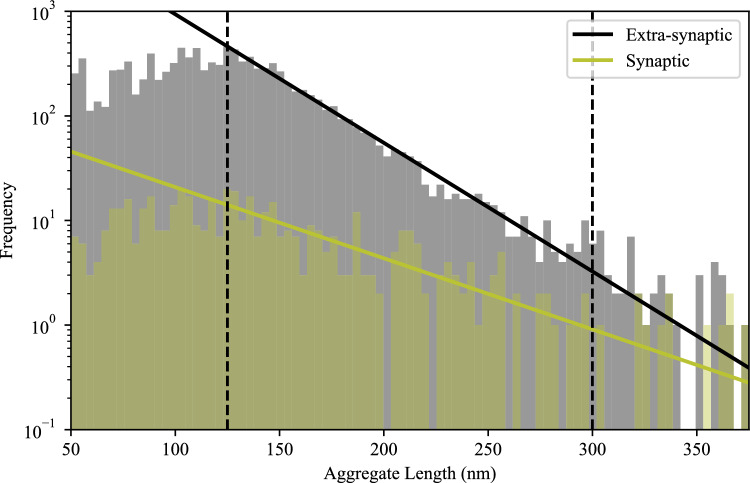
Histogram of the synaptic and extra-synaptic AT8 + tau aggregate length distributions (in nanometres (nm)) across all Braak stages. The solid lines show the maximum likelihood fit of Eq. (1) to the aggregate populations in each region. The two dashed lines show the upper and lower bounds of the fitted region

Following these control experiments to verify the SynPull results, we characterised the extra-synaptic AT8 + tau aggregates also detected by SynPull. The quantity of these aggregates was much higher at Braak stage 6 compared to stage 3, following a smaller change between stages 0 and 3 (Fig. 1j). However, the length of the extra-synaptic AT8 + tau aggregates did not show an AD stage-related difference and remained constant with a mean of approximately 135 nm (Fig. 1k). Similar to the synaptic aggregates, the extra-synaptic AT8 + tau aggregates were also mostly round, with even smaller eccentricity values compared to synaptic aggregates (Fig. 1e). Interestingly, while the fraction of long and elongated aggregates was higher for the extra-synaptic aggregates in the control cases (6% synaptic vs 13% extra-synaptic), this trend reversed in AD with 7–8% of the extra-synaptic tau aggregates longer than 150 nm in length with an eccentricity greater than 0.9 (Fig. 1g). Taken together, these results show that the number of small tau aggregates outside the synapse increases at later stages of AD, but no significant changes in aggregate length occur. This contrasts with the higher levels of synaptic aggregates at early stages, along with longer aggregates at later stages, suggesting that the synapse may be a more susceptible region of aggregation [22]. This was also shown by analysis of the aggregate length distribution. Both the synaptic and extra-synaptic aggregate length distributions were consistent with a geometric decay, which results from the balance of elongation and removal processes [13]. However, the synaptic aggregate length distribution decays more slowly with increasing length, indicating that the balance of elongation and removal processes inside the synapse is shifted more in favour of aggregation. We quantified the ratio of removal rate to elongation rate and found this to be reduced by 45 ± 6% for aggregates from synapses compared to the extra-synaptic ones (Fig. 2). While the majority of the AT8 + tau aggregates in AD are round, there is a greater fraction of elongated aggregates in the synapse than in the extra-synaptic fraction, showing distinct tau aggregate patterns between different parts of the neurons. The low concentration of elongated aggregates and abundance of small aggregates, which have been associated with local inflammatory responses [47], suggest that local inflammation may play a key role. To further study this, we looked at synaptic regulators of microglia activation along with markers of synaptic function.

### Synaptic tau becomes multi-phosphorylated and co-localises with functional markers

After showing the presence of AT8 + tau aggregates in the synapse at early stages of AD that are mostly non-elongated, we investigated the physiological and pathological outcomes that correlate with this accumulation. Studying individual synaptosomes and co-localising the AT8 + tau signal with T181-positive tau at the single-aggregate level, as well as determining the presence of exposed phosphatidylserine (“eat me” signal), CD47 (“don’t eat me” signal), and synaptogyrin-3 in synaptosomes containing AT8 + tau (see Supplemental Figs. 7 and 8 for representative images), we were able to explore how synaptic tau aggregation alters synaptic function and pruning, which are potentially contributing to cognitive deficits in AD. In order to do this, we determined 4 parameters: (1) the total amount of signal in the FoV, (2) the percentage of the synaptosomes carrying the signal ([Synaptosomes with Signal) / All Synaptosomes]*100), which estimates the marginal probability of observing the signal, (3) the percentage of the synaptosomes carrying the signal as well as AT8 out of the number of synaptosomes carrying AT8 ([Synaptosomes with Signal + AT8 + tau) / Synaptosomes with AT8 + tau]*100), which estimates the conditional probability of observing the signal given the presence of AT8 + tau, and (4) the percentage of the synaptosomes carrying the signal as well as AT8 out of the total number of synaptosomes ([Synaptosomes with Signal + AT8 + tau) / All Synaptosomes]*100), which estimates the joint probability of observing both AT8 + tau and the signal. It should be noted that these analyses were done using diffraction-limited imaging; as such, while the trends agree with the *d*STORM data presented above in terms of aggregate count, the exact numbers are not comparable between the methods. Based on these data, we then used Bayesian inference [4] to estimate the probabilities of co-occurrence of each type of signal with AT8 + tau, and compared this to the probability of occurrence of the signal regardless of the presence of AT8 + tau. This allowed us to determine if the presence of AT8 + tau leads to a changed probability of observing the signal, and how this correlation varies over disease stages.

**Fig. 3:**
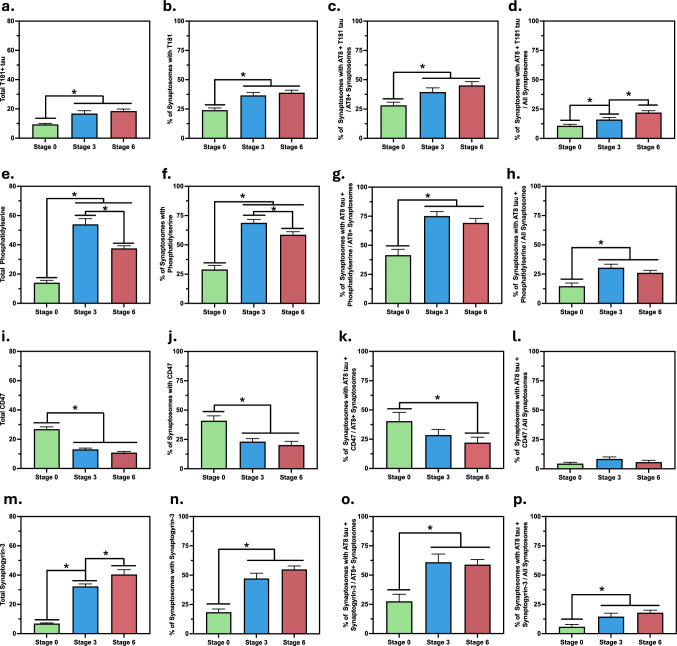
Pathology-associated markers measured inside the synaptosomes and co-localised with AT8-positive tau aggregates. (**a/e/i/m**) Total number of T181-positve tau (**a**), phosphatidylserine (**e**), CD47 (**i**), and synaptogyrin-3 (**m**) per field of view (FoV). (**b/f/j/n**) Synaptosomes carrying a T181-positve tau (**b**), phosphodiesterase (**f**), CD47 (**j**), and synaptogyrin-3 (**n**). (**c, g, k, o**) Percentage of synaptosomes carrying a T181-positve tau (**C**), phosphatidylserine (**g**), CD47 (**k**), and synaptogyrin-3 (**o**) and AT8-positive tau (co-localised in the same synaptosome), as a percentage of synaptosomes with AT8-positive tau. (**d, h, l, p**) Percentage of synaptosomes carrying a T181-positve tau (**d**), phosphodiesterase (**h**), CD47 (**l**), and synaptogyrin-3 (**p**) and AT8-positive tau (co-localised in the same synaptosome), as a percentage of all synaptosomes. Date in are represented as Mean ± SEM and differences are calculated using 95% confidence intervals (CI) with a CI not including 0 is considered significant, indicated with an asterisk

While the total T181-positive tau signal in the sample (Fig. 3a) and the fraction of synaptosomes with T181-positive tau (Fig. 3b) were both significantly higher in the Braak stage 3 samples compared to the controls, neither value was further elevated at stage 6. Similarly, multi-phosphorylation at AT8 and T181 as a fraction of synaptosomes with AT8 + showed a significant difference only between stages 0 and 3 (Fig. 3c), however the synaptosomes carrying multi-phosphorylated aggregates as a fraction of all synaptosomes (with or without AT8 + tau), showed a significant increase at stage 3 from stage 0 and a further increase at stage 6 (Fig. 3d). Together, these results show that tau phosphorylated at T181 increases early in AD and multi-phosphorylated tau is increased in disease and exists in more synapses as the disease progresses.

**Fig. 4:**
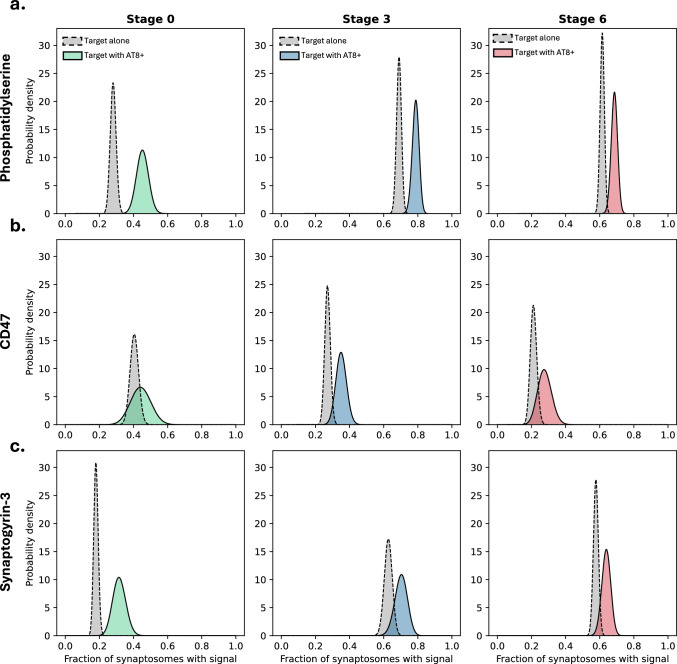
Probability to encounter synaptic markers and its correlation with AT8 state: Bayesian inference was performed to estimate the probability of encountering each of the synaptic markers (**a**) phosphatidylserine, (**b**) CD47, and (**c**) synaptogyrin-3. The posteriors are shown for encountering the signal regardless of AT8 state, i.e., the marginal probability (grey curves), and for encountering the signal if the synaptosome is AT8 positive, i.e., the conditional probability (coloured curves). A clear separation of the posteriors implies a significantly increased probability of encountering the signal in AT8 positive synaptosomes, as can be seen across stages for phosphatidylserine

Then, we investigated the “eat me” signal phosphatidylserine, which contributes to microglial synaptic pruning when exposed on the extracellular side of the cell membrane [63]. By using an antibody that specifically recognises exposed phosphatidylserine, we showed that while the total phosphatidylserine levels were higher at stage 3 compared to stage 0, they were lower at stage 6 compared to stage 3 (Fig. 3e). Similarly, the fraction of synaptosomes with exposed phosphatidylserine was also higher at stage 3 compared to both stages 3 and 6 (Fig. 3f). Together, these results indicate an early increase in signals for microglial synaptic pruning in AD, which decrease in the later disease stages. When we co-localised this “eat me” signal with AT8 + tau in the synaptosome, the percentage of co-localisation within the synaptosomes with AT8 + tau was significantly higher at stage 3, compared to stage 0, but did not further change at stage 6 (Fig. 3g). Similarly, the synaptosomes with the co-localisation of phosphatidylserine and AT8 + tau as a percentage of all synaptosomes was higher at stage 3 but did not show any further changes at stage 6 (Fig. 3h). We then used Bayesian inference, which demonstrated a clear correlation between the presence of AT8 + tau and the presence of phosphatidylserine, with a significantly higher probability of observing the signal when AT8 + tau was present, across all stages of the disease. As demonstrated by the very small overlap of the posterior probabilities in Fig. 4a, the difference was significant across all stages. Specifically, the probability that AT8 + tau has an effect on the presence of an eat-me signal was calculated as 99.9% in Braak stage 0, 99.1% in stage 3 and 85.1% in stage 6. Collectively, these results suggest an increase in phosphatidylserine exposure early in AD, possibly regulated by synaptic tau aggregation through interaction with flippases regulating the membrane localisation of phosphatidylserine [64] or by directly interacting with phosphatidylserine [44]. However, this is not a progressive condition as the levels are not further increased in later AD, suggesting additional mechanisms may be contributing to synaptic loss at later disease stages.

Following the “eat me” signal, we focussed on the “don’t eat me” signal CD47 in terms of its relationship with AT8 + tau at the single-synapse level. While the total CD47 levels in the samples were significantly reduced at Braak stage 3 compared to stage 0, there was no difference between stages 3 and 6 (Fig. 3i). In parallel to total CD47, the percentage of synaptosomes carrying the “don’t eat me” signal was also reduced significantly by stage 3, without any further reduction at stage 6 (Fig. 3j). When the co-localisation of AT8 + tau and CD47 within synaptosomes with AT8 + tau was compared between the Braak stages, no difference was seen between stage 0 and stage 3 or stages 3 and 6, yet the fraction of synaptosomes with CD47 and AT8 + tau co-localisation was significantly lower in stage 6 AD brains compared to the control brains (Fig. 3k). Meanwhile, the fraction of synaptosomes with the co-localisation of CD47 and AT8 + tau within all synaptosomes did not show any significant difference between disease stages (Fig. 3l). Collectively, these results show that while the expression of CD47 is reduced in AD in a progressive manner, the co-localisation of this “don’t eat me” signal and AT8 + tau is a rare event in the synaptosomes regardless of AD status. This was also evident from the strong overlap of posterior probability distributions in Fig. 4b, as the presence of AT8 + tau correlated only weakly with CD47, with the probability that AT8 + tau impacts the presence of CD47 in the same synaptosome was calculated as 10.9% in Braak stage 0, 44.7% in stage 3, and 16.7% in stage 6, suggesting that pathological tau-induced loss of CD47 at the single-synapse level may not be a primary mechanism of synaptic loss in AD.

Lastly, we quantified synaptogyrin-3 -which has been reported to mediate the binding of tau aggregates to neurotransmitter vesicles and cause pre-synaptic dysfunction[49] and investigated its co-localisation with AT8 + tau in the synaptosomes. Total synaptogyrin-3 levels were significantly higher in Braak stage 3 compared to stage 0 and in stage 6 compared to stage 3 (Fig. 3m). Meanwhile, the fraction of synaptosomes containing synaptogyrin-3 were elevated at stage 3 but did not further change at stage 6 (Fig. 3n). Similarly, when compared between the Braak stages, the percentage of synaptosomes with a co-localisation of synaptogyrin-3 and AT8 + tau, within the synaptosomes with AT8 + tau also showed an increase in stage 3 yet did not differ between stages 3 and 6 (Fig. 3o). When the percentage of synaptosomes with co-localisation of synaptogyrin-3 and AT8 + tau was calculated within all synaptosomes, once again increased levels were observed at stage 3 without further changes in stage 6 (Fig. 3p). Collectively, these results show an early increase in synaptogyrin-3 in AD, contributing to synaptic dysfunction. According to Bayesian inference, the probability of observing synaptogyrin-3 was increased in AT8 + tau containing synaptosomes. However, a strong correlation was observed only at stage 0, with weaker correlations at the later stages (Fig. 4c). Specifically, the probability that AT8 + tau has no effect on the appearance of Synapotgyrin-3 was 97.0% in stage 0, 27.6% in stage 3, and 29.6% in stage 6.

## Discussion

The role of synaptic tau aggregation in the morphological and (patho)physiological alterations in synapses is an active area of research [12, 65, 67]. While physiological presence and activity of tau have been reported in the synapse [58], it has also been shown that oligomeric Aβ can initiate the pathological translocation and aggregation of tau to the synapse leading to deficits [20]. Synaptic tau aggregates can interact with pre-synaptic vesicles to cause neuronal communication deficits [75] and alter the synaptic proteome [14], leading to synaptic dysfunction and loss, chiefly regulated by microglia [70]. Intriguingly, it was recently shown that in AD patients who showed behavioural symptoms of dementia and brains showing Braak stages 3 and 5 pathology, small tau aggregates are present in the synapses to a greater extent compared with patients with the same neuropathological features (same Braak staging) who were resilient to dementia symptoms [66], suggesting that the synaptic localisation of small tau aggregates is a major driver of cognitive decline in AD. In order to further investigate this disease mechanism, we studied synaptic tau aggregates in post-mortem human pre-frontal cortex samples at Braak stages 0, 3, and 6 using SynPull, which is a method we have developed [31] that allows single-molecule investigation in individual synaptosomes. We have also quantified the co-localisation of synaptic markers associated with pathology and protection, to understand the patho-physiological outcomes of the synaptic small tau aggregate accumulation.

It has previously been suggested that supersaturated proteins are enriched at synapses, leading to cell vulnerability in AD [22]. Here, we used SynPull with *d*STORM super-resolution imaging to quantify and characterise the synaptic and extra-synaptic AT8 + tau aggregates in the same pre-frontal cortex samples. While the synaptic aggregates showed an early increase in quantity by stage 3, their numbers were not significantly higher at stage 6, yet the fraction of synapses containing aggregates and the size of the aggregates inside the synaptosomes continued to grow by stage 6, showing continuous aggregation throughout the progression of AD. On the other hand, there was no size difference for the extra-synaptic tau aggregates between the Braak stages, but their quantity increased dramatically after stage 3, following a relatively miniscule increase at stage 3. This suggests that synaptic aggregation of AT8 + tau is not only an early event in AD, but it also precedes the pathological aggregation seen in the rest of the neuron. While this may be due to the active translocation of tau to the synapse promoted by the Aβ aggregates [20], oligomeric tau aggregates in the synaptosomes were also found to be hyperphosphorylated and associated with ubiquitinated substrates and elevated proteasome components [67], suggesting that the post-translational modifications of tau and a reduction in its aggregate removal rate would also contribute to this early synaptic aggregate accumulation. Strikingly, only a small portion of the AT8 + tau aggregates in the synaptic and extra-synaptic fractions were longer than 150 nm in length and showed high eccentricity, suggesting the lack of an elongated morphology (Fig. 1f), but the concentration of these species was higher in the synaptic fraction of AD cases (around 15%). By comparing the length distribution of synaptic and extra-synaptic aggregates, we were able to quantify the relative rate of aggregate removal compared to aggregate growth. We found that the longer aggregates encountered in the synapse indicate that the relative rate of removal there is approximately half the rate for extra-synaptic aggregates, providing an explanation for earlier aggregation in the synapse and the early synaptic deficits in AD. Although it should be noted that the sources of the extra-synaptic aggregates are not only the rest of the neuron, as they may also be harvested from glial cells or the extra-cellular space.

Spreading of tau pathology through functionally connected brain regions in a “prion-like” manner has been suggested [10, 12, 62] and fibrillar tau aggregates around 170 nm in length have been demonstrated to be the most seeding-competent species [24, 27, 29, 72]. On the other hand, the smaller oligomeric tau aggregates are shown to promote local inflammation driven by microglia [47]. Our results do not rule out synaptic spreading of tau pathology through functional connectivity and seeding as a relevant mechanism as we do observe a small number of aggregates associated with this disease mechanism, specifically in the AD cases. However, the fact that the majority of the aggregates are small and non-elongated supports the idea that local microglia response to synaptic tau aggregates and the resulting inflammation play a key role in driving AD pathology. This interpretation is further supported by the association of microglia-response related signals with tau aggregates in individual synapses, as further discussed below.

To check the phosphorylation state of the synaptic tau aggregates, we quantified the co-localisation of AT8- and T181-positive tau signal in individual synaptosomes. The fraction of synaptosomes with T181-positive tau, as well as synaptosomes with co-localisation as a fraction of all synaptosomes with AT8 + tau, was higher in Braak stage 3, yet did not change at stage 6, showing that multi-phosphorylation of tau in the synapse is indeed an early event in AD. Nevertheless, the AT8 and T181 co-localisation showed a further increase at stage 6, showing that the newly formed aggregates inside the synapses at later stages of AD are more likely to be multi-phosphorylated. These results agree with previous findings of hyperphosphorylated tau aggregates being present in synaptosomes in AD [67].

After showing the presence of tau aggregates in synaptosomes as an early event in AD pathogenesis, we investigated the patho-physiological outcomes of this accumulation. A number of proteins have been associated with synaptic dysfunction and synaptic pruning, including phosphatidylserine, CD47, and synaptogyrin-3. Phosphatidylserine is also known as the “eat me” signal, as it mediates microglial synaptic pruning, regulated by TREM2 [63]. Our previous work has shown that neurons with tau filaments expose phosphatidylserine before getting phagocytosed by microglia [8] and spines that become overactive through oligomeric Aβ stimulation get pruned by microglia upon externalising phosphatidylserine [59]. On the other hand, CD47 functions as a “don’t eat me” signal, protecting synapses from microglia-mediated pruning during development [41]. Meanwhile, pre-synaptic co-localisation of tau with synaptogyrin-3 contributes to synaptic plasticity deficits along with working memory dysfunction and genetic silencing of synaptogyrin-3 ameliorates these symptoms in mouse models of tau pathology, without altering tau induced neuroinflammation [38]. We quantified these markers in individual synapses as well as their co-localisation with AT8 + tau, which allowed us to determine how the probability of the presence of these signals is altered by the presence of AT8 + tau.

It has previously been shown that tau oligomer-containing synapses in AD are eliminated by microglia and astrocytes [66]. In accordance with these findings, the phosphatidylserine signal showed a significant increase in early disease stages and Bayesian inference showed a higher chance for the presence of this signal in AT8 + tau-containing synaptosomes, even in non-AD brain samples and in late-stage AD, where the fraction of synaptosomes containing phosphatidylserine was reduced. Although a causal mechanism needs to be demonstrated further in future studies, this finding indicates a role for synaptic tau aggregate accumulation and “eat me” signals in tau-driven early synaptic loss in AD.

Through a similar mechanism, phosphatidylserine also works as an “eat me” signal during developmental synaptic pruning [63], counteracted by CD47, which protects the synapses [41]. Interestingly, increased CD47 levels were observed in the AD hippocampus, co-localising with synaptic tau aggregates [56]. While our analysis of the pre-frontal cortex samples showed a Braak stage-dependent decrease in overall CD47 levels, there was a weak trend of increase in the presence of CD47 in synaptosomes that are AT8 + tau at Braak stage 3. These findings suggest that while synaptic protective signals are reduced in AD, localising a “don’t eat me” signal in the affected synapses may be an attempt by the neurons to reduce synaptic loss during early stages, when tau-mediated synaptic loss becomes an active disease mechanism.

Similar to the “eat me” and “don’t eat me” signals, synaptogyrin-3 was also increased in AD, yet Bayesian inference did not show a meaningful effect of AT8 positivity on increasing the presence of synaptogyrin-3 in the disease samples. Synaptogyrin-3 mediates the binding of tau to pre-synaptic vesicles [49], which causes deficits in vesicle mobility and neurotransmitter release [75]. This suggests a pathological role of synaptogyrin-3 in relation to the synaptic presence of tau, by mediating its interaction with synaptic vesicles. As such, the presence and increase of synaptogyrin-3 in the synapse may be an independent event from synaptic tau aggregation and even precede it. In agreement with this, increased synaptic synaptogyrin-3 levels, compared to stage 0, were observed at stages 3 and 6. Since this increase was not linked to tau aggregation, there may be another, upstream mechanism. Activation of nuclear receptor 4A2 (NR4A2, also known as NURR1) was shown to increase synaptogyrin-3 expression [42] and Moon et al., [50] have shown a significant positive correlation between NR4A2 expression and Aβ aggregation in the 5xFAD mouse model of Aβ accumulation. This association between Aβ, NR4A2, synaptogyrin-3, and tau aggregates proposes an AD mechanism, with which Aβ aggregation leads to higher synaptogyrin-3 levels, which contribute to tau mediated synaptic dysfunction. Indeed, we have shown (Supplemental Fig. 4b) an early increase in stage 3—preceding an increase in total soluble Aβ levels, further supporting the presence of this AD mechanism.

### Conclusions, limitations, and future directions

In this study, we investigated the synaptic accumulation of small tau aggregates and their possible role in synaptic dysfunction through various markers in post-mortem AD pre-frontal cortex samples at different Braak stages. While the synaptic accumulation of AT8 + tau aggregates was already apparent at early stages of AD, the morphology of these aggregates continued to evolve by Braak stage 6, showing that synaptic tau aggregation remains a disease process throughout the pathogenesis of AD. Importantly, the presence of small-diffusible Aβ aggregates correlated with synaptic tau pathology, suggesting possible links, such as synaptogyrin-3-mediated tau binding to synaptic vesicles, which should be further investigated in future studies. On the other hand, synaptic pruning seems to be directly mediated by synaptic tau aggregation, by an increase in phosphatidylserine, as the presence of this signal was predicted by Bayesian inference to be directly linked to synaptic AT8 + tau aggregates. Meanwhile, there seems to be an attempt to reduce this synaptic loss, with an increase in CD47 signalling, which works as a “don’t eat me signal” during neurodevelopment. It is noteworthy that the “eat me” and “don’t eat me” signals, which are important for brain development at early stages of life, are also involved in AD—much later on, showing that AD hijacks the homeostatic physiology of the nervous system during its pathogenesis, rather than using novel mechanisms. Importantly, all of the pathological changes we studied here were most prominent at Braak stage 3, before an exponential increase in extra-synaptic tau aggregate levels and tau tangle pathology in the pre-frontal cortex, supporting that early synaptic deficits precede neuronal dysfunction and loss in AD, and are mediated by small-diffusible aggregates rather than much larger plaques and tangles. While some aggregates longer than 150 nm in length with fibril-like morphologies were detected, the very low concentration of these aggregates relative to the shorter and rounder species indicates that these smaller aggregates, and thus the related microglial response, play a more prominent role in synaptic dysfunction during the early stages of AD.

While these results are valuable for indicating the role of nanoscopic tau aggregates in synaptic dysfunction in AD, there are a number of limitations of our work that needs to be acknowledged. The synaptosome extraction protocol used in this study predominantly harvests the pre-synaptic compartment with parts of the post-synapse attached in some cases [26, 31], and thus the post-synaptic tau aggregates cannot be characterised. Since the post-synaptic accumulation of tau has been shown in AD [12] and the synaptic spread of tau in functionally connected neurons has been suggested as a disease mechanism [62], the inability to study post-synaptic tau aggregates and their co-localisation with markers associated with post-synaptic dysfunction is a limitation of this work. Moreover, SynPull and STED imaging were done on the same tissue samples, which contained multiple layers of the orbitofrontal cortex. As such, cortical layer-dependent differences were outside the scope of this work. Even though the SynPull results were supported using additional microscopy and SIMOA-based methods, due to the differences in sample preparation and limits of resolution between these methods, the exact quantity and size values are only comparable within each method. It should also be noted that factors such as the post-mortem interval and sample preparation with fixation steps may have an impact on the aggregates. Since tau leads to synaptic loss, there is a survival bias, through which the (patho)physiology of the synapses that remained intact during the time of death of the AD patient may differ from those that were lost throughout the disease process. Lastly, since we only studied post-mortem human brain samples, our results, while highly valid, remain correlational. As such, causational inferences on the role of tau aggregates for the synaptic accumulation of pruning signals cannot be made. Similarly, while the increase in phosphatidylserine with tau can be interpreted as a mechanism for synaptic loss, it can also be due to dysfunctional microglial pruning, leading to the accumulation of synapses with “eat me” signal. Future work on animal and cellular models with direct manipulation of tau as well as other markers that we have studied here will be beneficial for identifying the direct and temporarily causal links between synaptic tau aggregation, pathology, and cognitive decline. Nevertheless, the results we present here provide strong support for the early synaptic aggregation of multi-phosphorylated nanoscopic tau aggregates and their involvement in microglia-mediated synaptic pruning by altering the levels of signalling markers.

## Ethical approval

Human post-mortem brain tissue was acquired from the Cambridge Brain Bank (Cambridge University Hospitals). The Cambridge Brain Bank is supported by the NIHR Cambridge Biomedical Research Centre (NIHR203312). We gratefully acknowledge the participation of all our patients and control volunteers.

## Supplementary Information

Below is the link to the electronic supplementary material.

Supplementary file1 (PDF 25975 KB)

## Data Availability

Data collected during these experiments will be made available upon reasonable request from the corresponding author. Novel code generated for the analysis of STED images can be accessed at [https://github.com/lemur01/AggregateStats].
