# Supplementary material for: Early synaptic pathology is associated with small tau aggregates in Alzheimer’s disease
Source: Acta Neuropathol. 2026 Jan 23;151(1):7. doi: 10.1007/s00401-026-02977-9 (PMC12830486; doi:10.1007/s00401-026-02977-9)
Supplement: Supplementary file 1 — Supplementary file1 (PDF 25975 KB) [file 401_2026_2977_MOESM1_ESM.pdf]

Pre-frontal cortex **Medial-temporal lobe**

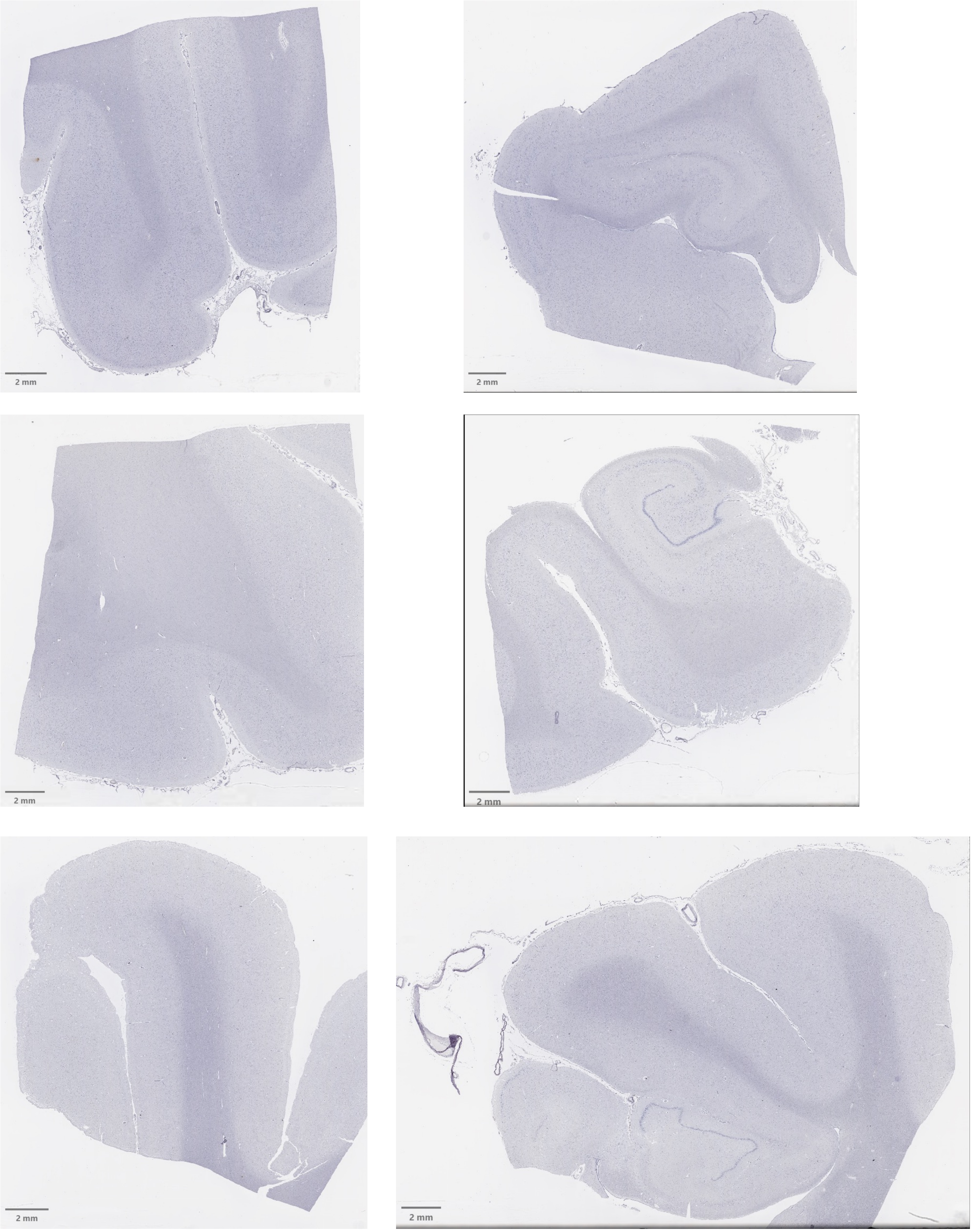

Supplemental Figure 1. Representative micrographs from pre-frontal cortex and medial-temporal lobe of control cases immune-stained with anti-phosphotau (Ser202, Thr205) antibody AT8, showing a lack of pathology in both regions, in accordance with Braak stage 0 pathology[7]. Scale bar is 2 millimetres.

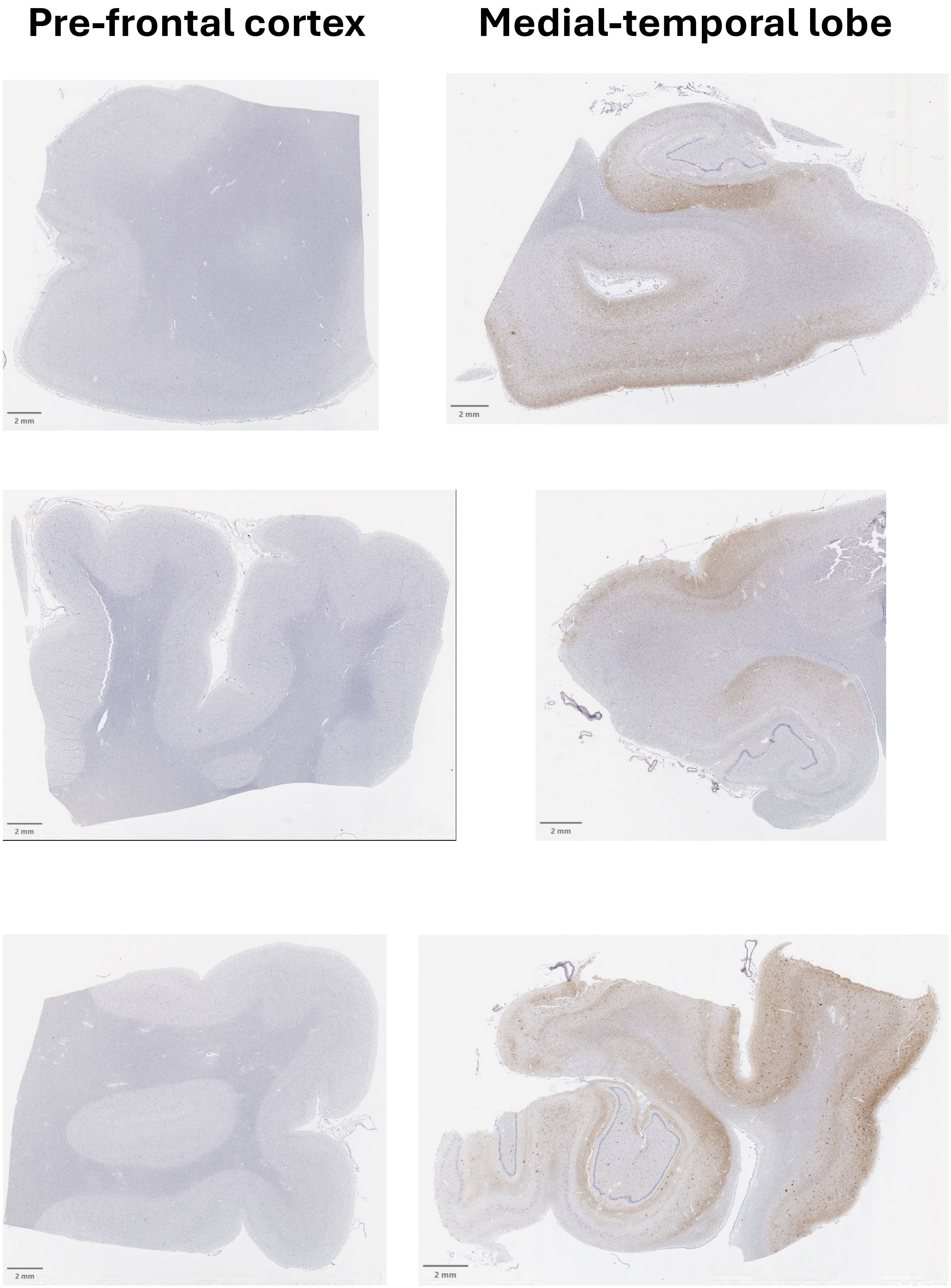

Supplemental Figure 2. Representative micrographs from pre-frontal cortex and medial-temporal lobe of early Alzheimer's disease cases immune-stained with anti-phosphotau (Ser202, Thr205) antibody AT8, showing a lack of pathology in the pre-fontal cortex, with pathological staining in the medial-temporal lobe, in accordance with Braak stage 3 pathology[7]. Scale bar is 2 millimetres.

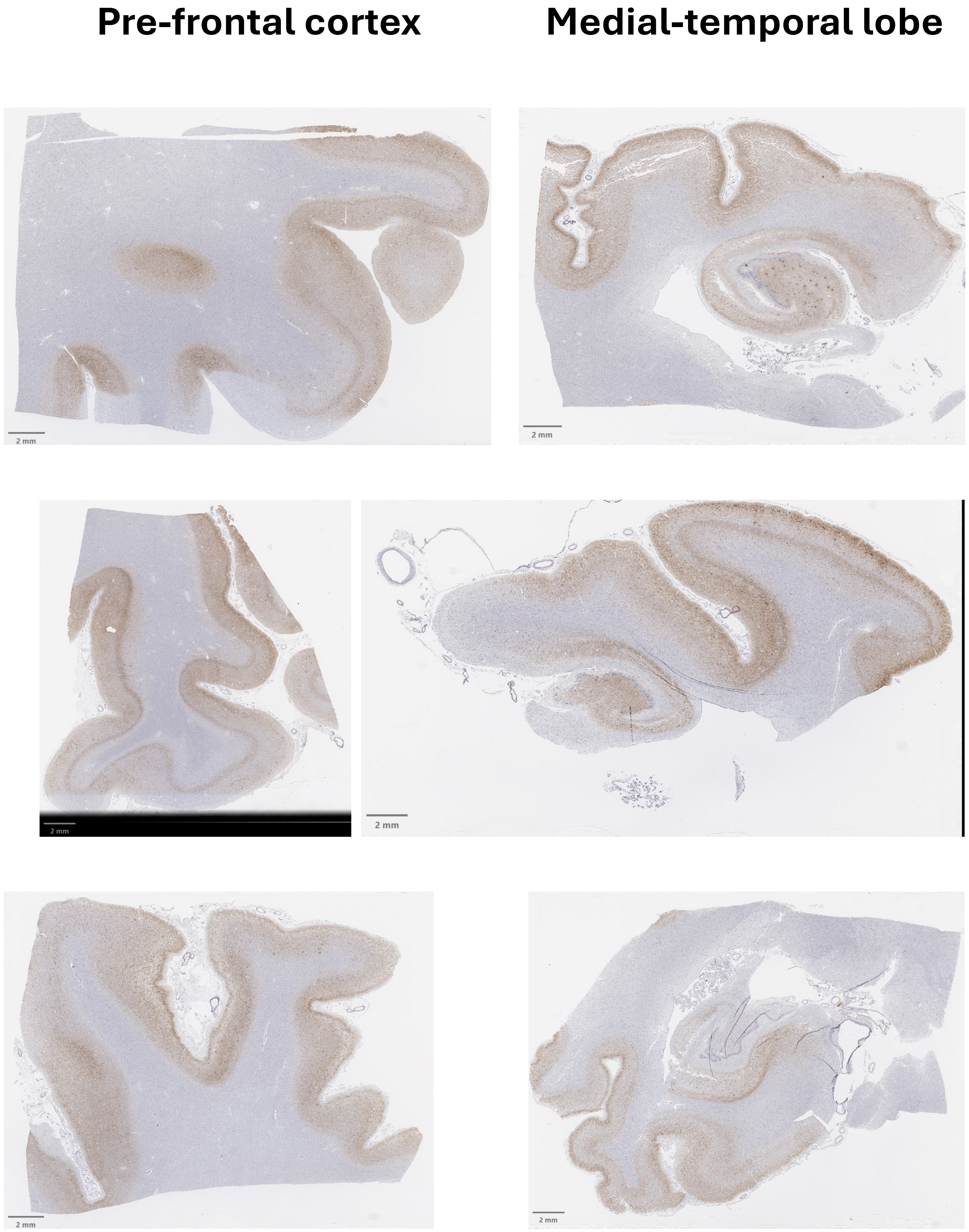

Supplemental Figure 3. Representative micrographs from pre-frontal cortex and medial-temporal lobe of advanced Alzheimer’s disease cases immune-stained with anti-phosphotau (Ser202, Thr205) antibody AT8, showing pathological staining in both regions, in accordance with Braak stage 6 pathology[7]. Scale bar is 2 millimetres.

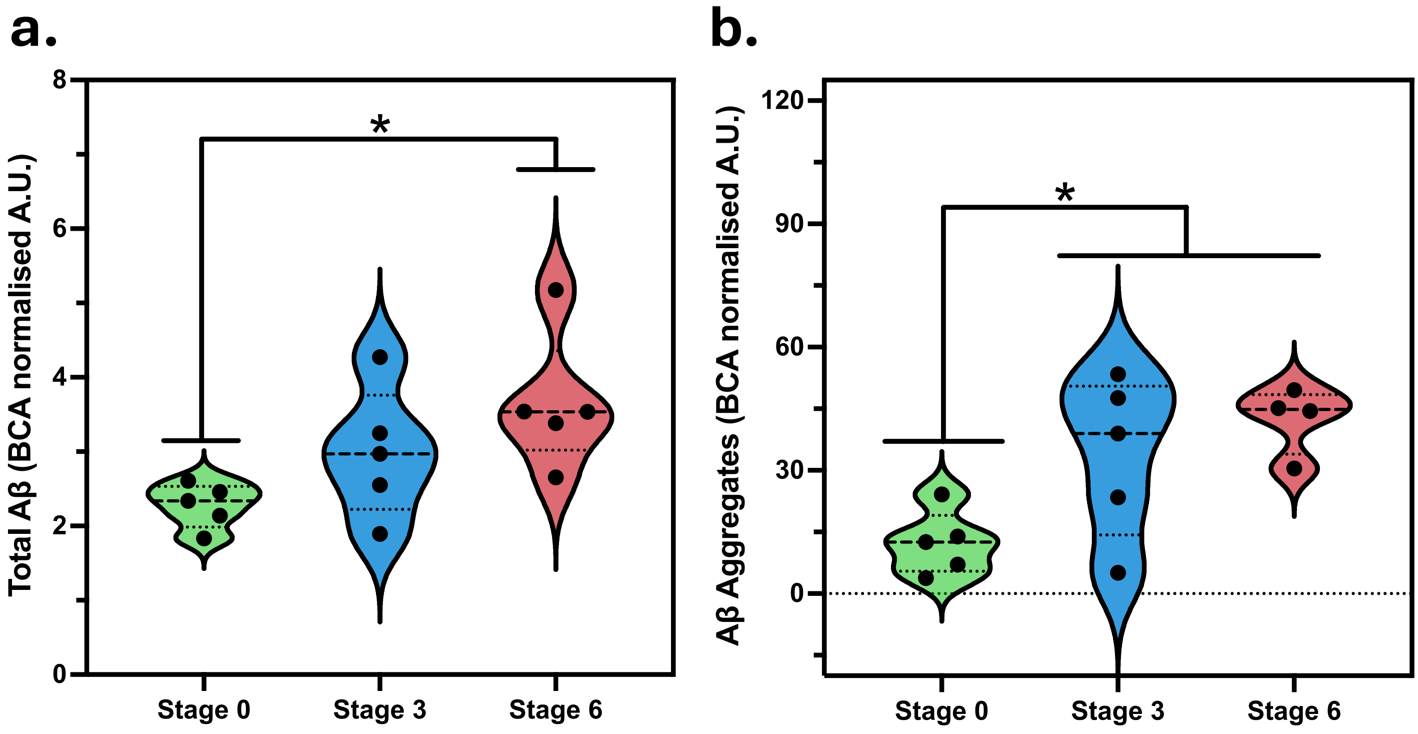

Supplemental Figure 4. Soluble beta-amyloid concentration in the pre-frontal cortex samples. (**a**) Total (monomer and aggregate) concentration measured by ELISA and (**b**) aggregate concentration measured by SIMOA. Values are normalised using bicinchoninic acid assay (BCA) assay and presented in arbitrary units (A.U.). Individual data points represent different brain samples at each Braak stage and differences are calculated using 95% confidence intervals (CI) with a CI not including 0 is considered significant, indicated with an asterisk.

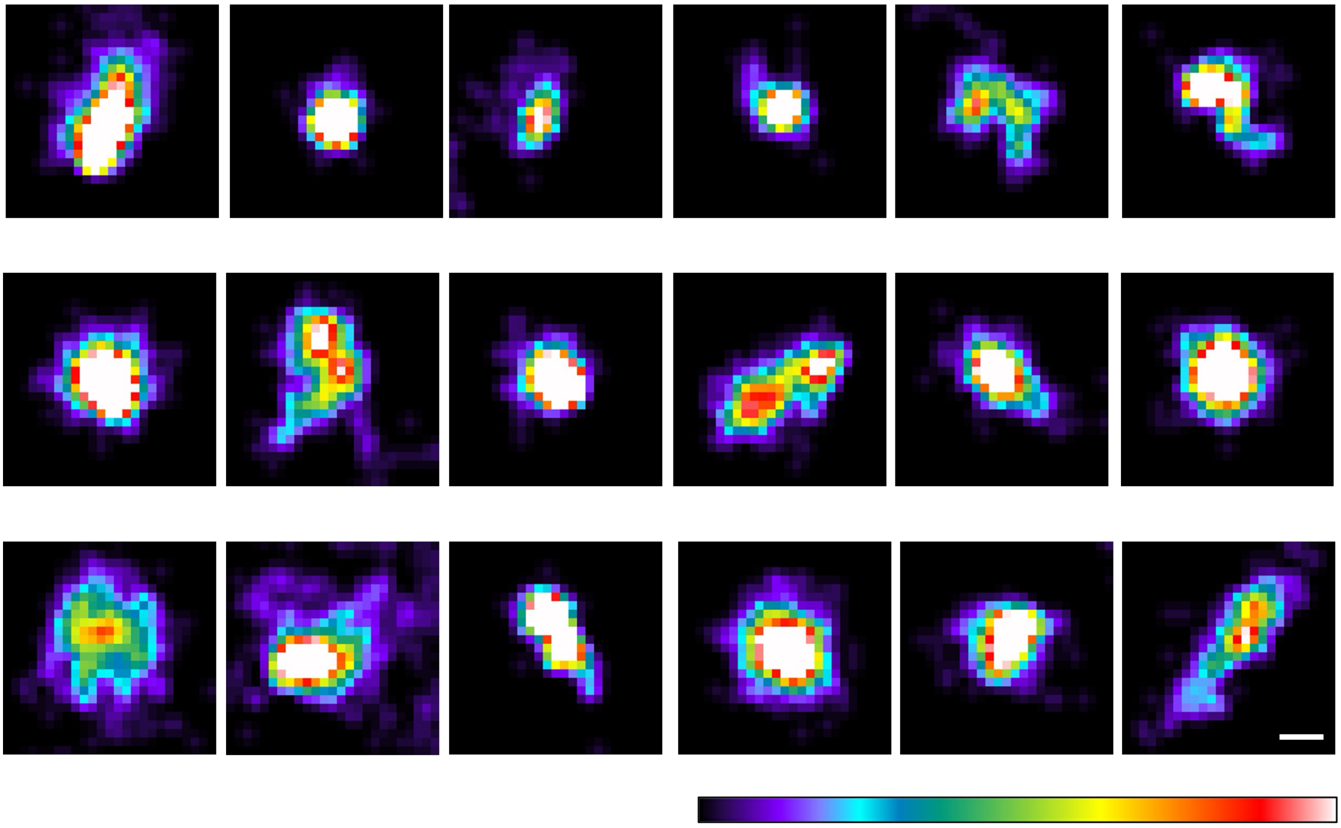

Supplemental Figure 5. Representative pseudo-colour images of AT8-positive tau aggregates from human brain samples acquired by *d*STORM microscopy and reconstructed by ACT software. The upper horizontal panel corresponds to Braak stage 0, the middle to stage 3, and the bottom to stage 6. The intensity is pseudo-colour coded from black (minimum) to white (maximum). Individual aggregates were segmented using MetaMorph software v7.10.5.476 (Molecular Devices)[3, 31, 33]. Scale bar is 50 nm.

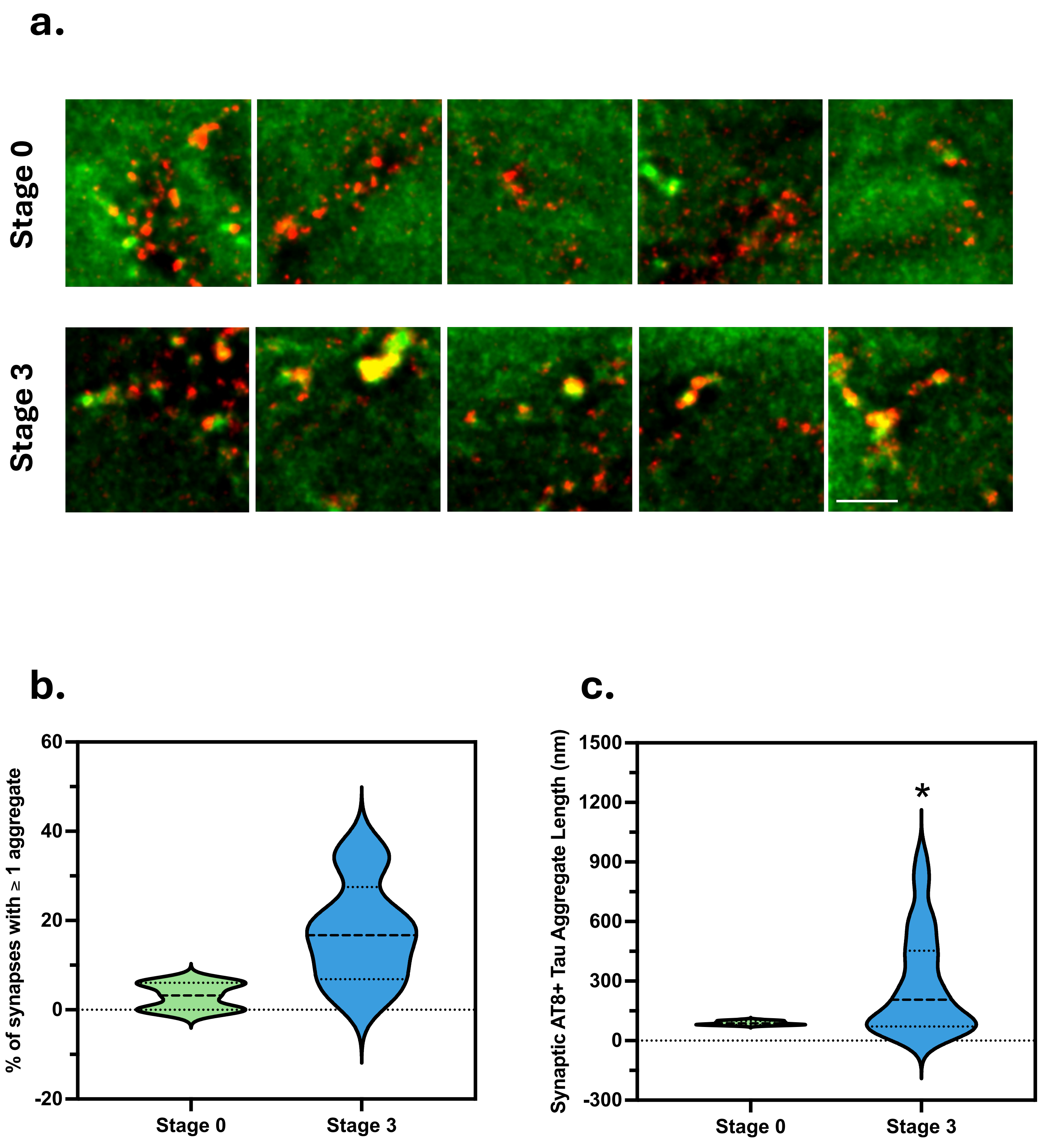

Supplemental Figure 6. Results from STED imaging. Pseudo-colour overlay showing nanoscale visualization of a section of human brain immune-stained for a marker for pre-synapse (Synapsin1/2) in red with AT8-tau (Ser202, Thr205) in green. The yellow indicates co-localization. The diffuse green staining is signal beyond the resolution limit of STED and was not used in the cluster analysis (see STED methods for further details). Scale bar is 1 µm (**a**). Percentage of synapses containing one or more AT8+ tau aggregate (95% CI = -0.21, 28.23; **b**) and average synaptic aggregate length (95% CI = 120.23, 277.36; **c**). Significance determined by 95% confidence intervals.

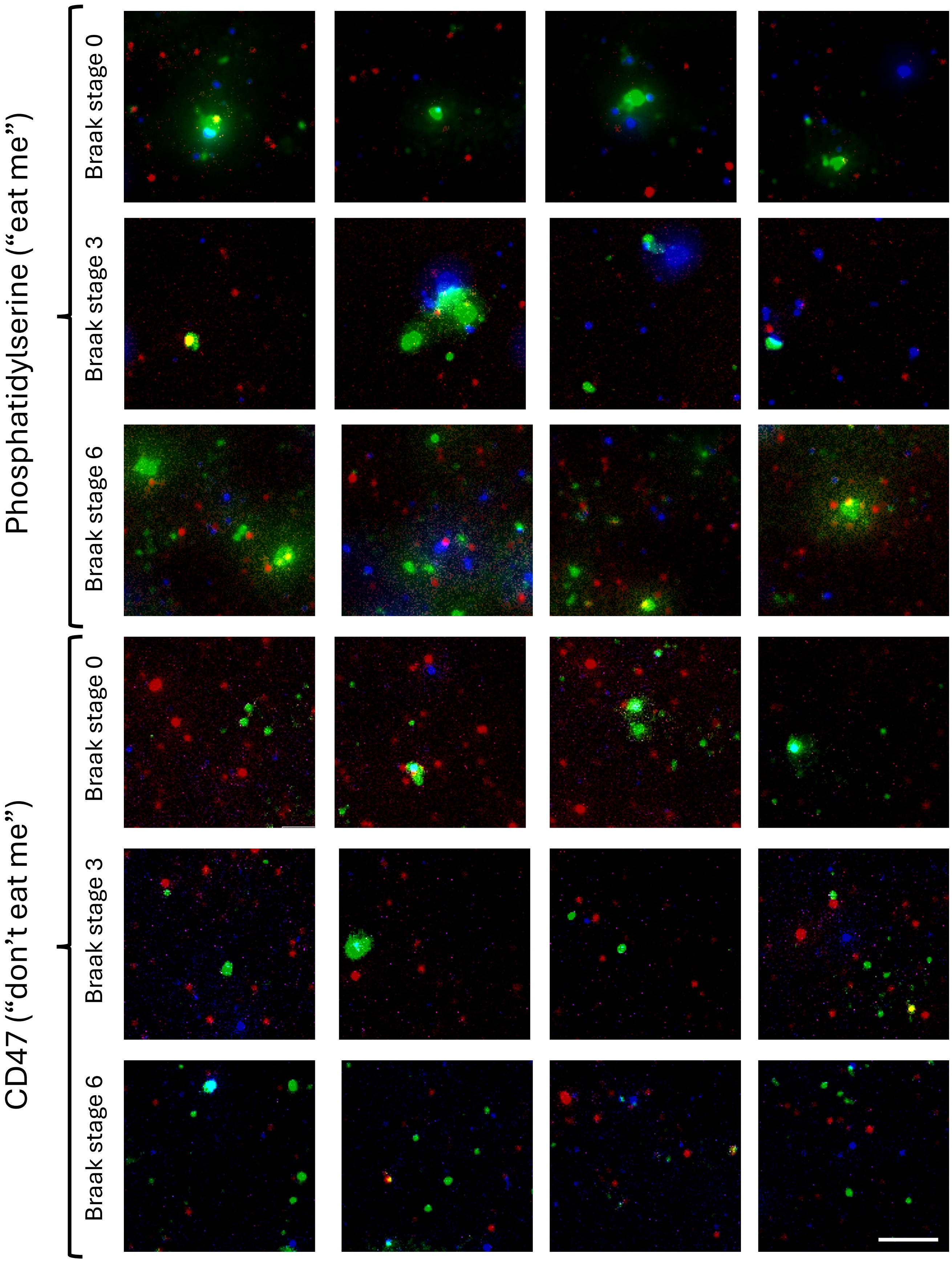

Supplemental Figure 7. Pseudo-colour fluorescence microscopy overlays of human brain samples processed with SynPull. In the first three horizontal panels, phosphatidylserine ("eat me") is shown in blue, the synaptosome CellMask in green, and AT8-positive phosphorylated tau (Ser202, Thr205) in red. The fourth through sixth panels show pseudo-colour overlays for CD47 ("don't eat me") in blue, the synaptosome cell mask in green, and AT8-positive tau in red. The first and fourth horizontal panels correspond to Braak stage 0, the second and fifth to Braak stage 3, and the third and sixth to Braak stage 6. Scale bar is 5 µm.

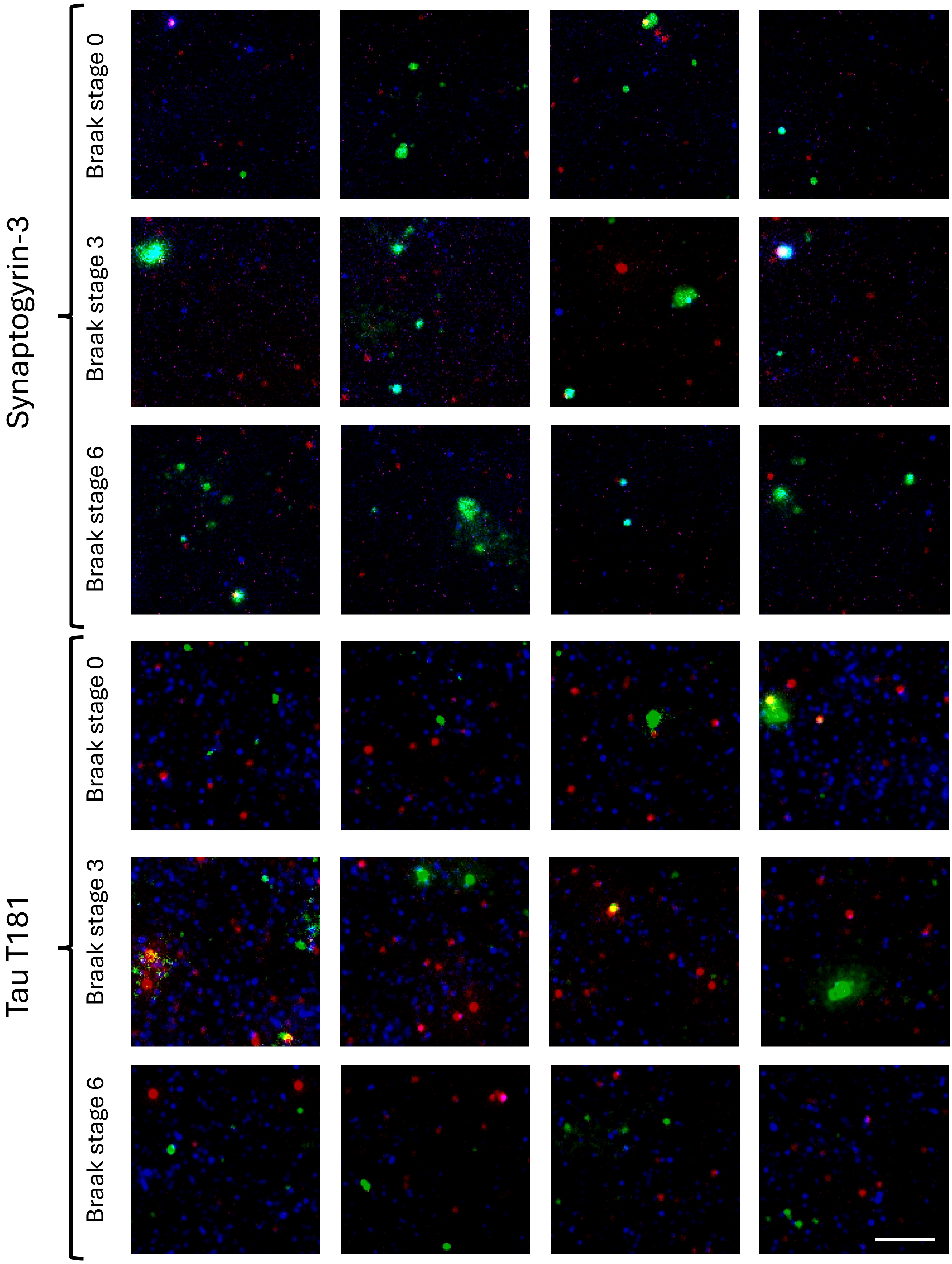

Supplemental Figure 8. Pseudo-colour fluorescence microscopy overlays of human brain samples processed with SynPull. In the first three horizontal panels, Synaptogyrin-3 is shown in blue, the synaptosome CellMask in green, and AT8-positive phosphorylated tau (Ser202, Thr205) in red. The fourth through sixth panels show pseudo-colour overlays for p-Tau T181 in blue, the synaptosome cell mask in green, and AT8-positive tau in red. The first and fourth horizontal panels correspond to Braak stage 0, the second and fifth to Braak stage 3, and the third and sixth to Braak stage 6. Scale bar is 5 μm.
